# The effects of quercetin on microRNA and inflammatory gene expression in lipopolysaccharide-stimulated bovine neutrophils

**DOI:** 10.14202/vetworld.2017.403-410

**Published:** 2017-04-18

**Authors:** Phongsakorn Chuammitri, Suphakit Srikok, Duanghathai Saipinta, Sukolrat Boonyayatra

**Affiliations:** 1 Department of Veterinary Biosciences and Public Health, Faculty of Veterinary Medicine, Chiang Mai University, Chiang Mai 50100, Thailand; 2 Dairy Cow Hospital, Faculty of Veterinary Medicine, Chiang Mai University, Mae On, Chiang Mai 50130, Thailand; 3 Department of Food Animal Clinic, Faculty of Veterinary Medicine, Chiang Mai University, Chiang Mai 50100, Thailand

**Keywords:** bovine neutrophil, gene expression, microRNA, quercetin

## Abstract

**Aim:**

To investigate gene expression of microRNA (miRNA) milieus (*MIRLET7E*, *MIR17*, *MIR24-2*, *MIR146A*, and *MIR181C*), inflammatory cytokine genes (interleukin 1β [*IL1B*], *IL6*, *CXCL8*, and tumor necrosis factor [*TNF*]), and the pathogen receptor toll-like receptor (*TLR4)* in bovine neutrophils under quercetin supplementation.

**Materials and Methods:**

Isolated bovine neutrophils were incubated with bacterial lipopolysaccharide under quercetin treatment or left untreated. Real-time polymerase chain reaction was performed to determine the expression of the miRNAs and messenger RNA (mRNA) transcripts in neutrophils.

**Results:**

Quercetin-treated neutrophils exhibited a remarkable suppression in *MIR24-2*, *MIR146A*, and *MIR181C* expression. Similarly, mRNA expression of *IL1B*, *IL6*, *CXCL8*, *TLR4*, and *TNF* genes noticeably declined in the quercetin group. Many proinflammatory genes (*IL1B*, *IL6*, and *CXCL8*) and the pathogen receptor *TLR4* had a negative correlation with *MIR146A* and *MIR181C* as revealed by Pearson correlation.

**Conclusions:**

Interaction between cognate mRNAs and miRNAs under quercetin supplementation can be summarized as a positive or negative correlation. This finding may help understand the effects of quercetin either on miRNA or gene expression during inflammation, especially as a potentially applicable indicator in bovine mastitis.

## Introduction

Bovine mastitis is an important health issue in dairy cattle, causing significant economic losses involving the dairy industry worldwide [1]. Mastitis in dairy cattle is usually caused by bacterial pathogens that invade the mammary gland and surrounding tissues of milk cows; *Staphylococcus aureus*, *Streptococcus agalactiae, Streptococcus uberis*, and *Escherichia coli* have been found to cause the disease by transmission from cow-to-cow and from the environment [2]. After the initiation of infection, polymorphonuclear leukocytes, especially neutrophils, travel to infected sites as a consequence of an innate immune process and may trigger inflammation by their accumulation. The inflammation of mammary tissues after the onset of infection can be manipulated by many cytokines and inflammatory mediators such as tumor necrosis factor-α (TNF-α), interleukin 1β (IL1β), IL8, and prostaglandin F2α [3].

The microRNAs (miRNA) are endogenous, non-protein coding, single-stranded RNAs of ~23 nucleotides long [4-6]. miRNAs are generated by various cell types and then secreted into extracellular spaces and fluids. Their presence has been confirmed in cows, and these miRNAs have been characterized and fully elucidated [7-11]. In human medicine, miRNAs have long been of particular interest for the study of disease versus normal health status [5,12]. The discovery of bovine miRNAs may lead to their application as biomarkers in the diagnosis of many diseases involving bovine health.

Quercetin, one of the most widely distributed flavonoids in the plant, is a flavonoid ubiquitously present in both the food sources and beverages. It is reported to be rich in many fruits, vegetables, herbs, nuts, seeds, stems, flowers, and plant-derived beverages such as tea and wine. Quercetin possesses a broad range of pharmacological properties including anti-inflammatory effects, antiproliferative effects (in tumor cells), and protective effects against oxidative stress. It exerts a strong antioxidant, anti-inflammatory, antimetastatic effect in various types of cancer, anticarcinogenic, antiviral, cardioprotective, estrogenic and antiestrogenic effects [13-18]. A study by Chuammitri *et al*. on the effects of quercetin (a plant flavonoid) on bovine neutrophils found that quercetin modulates many *in vitro* functions of these cells, but the effects of quercetin on gene expression related to miRNAs and proinflammatory genes have not yet been determined [16]. Studies on the effects of quercetin on gene expression in cows, specifically in the innate immune system, have been very limited, although the suppression of proinflammatory cytokines and genes involved in the inflammatory process, for example IL1β and TNF, in bovine immune cells has been documented in the literature [16]. Quercetin reduces the expression levels of genes and proteins directly or indirectly connected with inflammation and innate cellular functions [16,19-21].

This study was designed to investigate the gene expression of miRNAs (*MIRLET7E, MIR17, MIR24-2, MIR146A*, and *MIR181C*) and inflammatory cytokine genes (*IL1B, IL6, CXCL8*, toll-like receptor [*TLR4*], and *TNF*) in bovine neutrophils under supplementation of quercetin during the induction of inflammation by bacterial lipopolysaccharide (LPS).

## Materials and Methods

### Ethical approval

The animal experiments were approved by the Animal Care and Use Committee, Faculty of Veterinary Medicine, Chiang Mai University, Thailand (Ref. no. R4/2559).

### Animals, blood collection, and bovine neutrophil isolation

Healthy, non-pregnant adult Holstein-Friesian cows (*Bos taurus*) were used as blood donors. A total of 12 cows in two independent experiments, 6 cows each, were recruited into the study. All dairy cows were housed at local dairy farms in Mae On district, Chiang Mai, Thailand. About 40 ml of whole blood was collected by jugular venipuncture into a sterile syringe containing 10 ml of 1 X acid citrate dextrose solution for neutrophil isolation within 2 h after blood collection. Bovine neutrophil isolation was performed as previously described by Chuammitri *et al*. [16]. Isolated neutrophils were cultured in RPMI 1640 supplemented with 1% heat-inactivated fetal bovine serum (Thermo Fisher Scientific, Waltham, MA, USA). Cell viability was assessed by trypan blue exclusion and cytospin preparation.

### Quercetin

Stock quercetin solution (5 mM) was prepared by dissolving dry quercetin powder (Sigma-Aldrich, St. Louis, MO, USA) in 95% ethanol and then passing through a sterile filter. A working solution at a concentration of 50 µM was freshly prepared on the day of the experiment, protected from light, and stored at room temperature until use. The 50 µM concentration of quercetin showed no *in vitro* cytotoxicity to bovine neutrophils as in the previous report [16].

### Bovine neutrophil stimulation

Two million bovine neutrophils (2 × 10^6^ cells) were seeded in triplicate wells of a 24-well cell culture plate. Subsequently, cells were treated with 100 ng/mL LPS from *E. coli* O111:B4 (Sigma-Aldrich). Either 50 µM quercetin or phosphate buffered saline (PBS) was supplemented by the time of LPS stimulation. Plates were incubated for 60 min at 37°C in 5% CO_2_. After stimulation, cells from triplicate wells were combined, washed, and spun (400 ×*g*, 5 min). Stimulated bovine neutrophil cell pellets were resuspended in RNAlater^®^ (Thermo Fisher Scientific), according to the manufacturer’s instructions, to preserve RNA for further analysis.

### Messenger RNA (mRNA) and miRNA purification, and complementary DNA (cDNA) synthesis

Two fractions of RNA (small and large RNA) from bovine neutrophils were purified using a NucleoSpin miRNA kit (Macherey-Nagel, Bethlehem, PA, USA) according to the manufacturer’s recommendations. The isolation of small RNA (<200 nt) and large RNA (>200 nt) was performed simultaneously but eluted in two separate fractions. The eluted mRNAs were contained in a large RNA fraction, while the miRNAs were dissolved in another fraction.

The RNA concentration was quantified using a DU 730 ultraviolet/visible spectrophotometer equipped with a nanoVette microliter cell accessory (Beckman Coulter, Brea, CA, USA). cDNA was reverse-transcribed from a starting amount of 1 μg of total RNA with a Tetro cDNA Synthesis Kit (Bioline, Taunton, MA, USA). Samples were then stored at −20°C until further use.

### Bioinformatics and primer design

The primer design was based on a searchable database of published miRNA sequences and annotation of bovine miRNA information given by the miRBase release 21: June 2014 (http://www.mirbase.org/), and PubMed (http://www.ncbi.nlm.nih.gov/Pubmed/). The gene targets for respective miRNAs were predicted using TargetScan Human release 6.2: June 2012 (http://www. targetscan.org). The primers were designed from the pre-miRNA or stem-loop sequence of corresponding miRNA by placing the forward and reverse primers flanking the mature miRNA; miR-16a was used as a reference miRNA gene [22]. Primers were designed using Primer3Plus and synthesized by Macrogen, Seoul, South Korea. The sequences of the oligonucleotide primers used in this study are listed in Supplementary Tables-S1 and S2.

**Supplementary Table-S1:** Oligonucleotide primers for pre-miRNA/stem-loop miRNA genes.

miRNA	Accession no.	Forward primer (5′-3′)	Reverse primer (5′-3′)	Products (bp)
*MIRLET7E*	NR_031388	CCCGGGCTGAGGTAGGAGGT	CCTGGGGAAAGCTAGGAGGC	79
*MIR17*	NR_031371	GTCAGAATAATGTCAAAGTGCTT	GTCACCATAATGCTACAAGTGC	84
*MIR24-2*	NR_030968	CTCTGCCTCCCGTGCCTACT	CCTGTTCCTGCTGAACTGAGC	72
*MIR146A*	NR_031031	CCCATGTGTATCCTCAGCTTT	AGGATGATAGAGATATCCCAGC	99
*MIR181C*	NR_031372	TTGCCAAGGGTTTGGGGGAAC	GGCAGTTCCAGGCCTCGGG	97
*MIR16A*	NR_031032	GTCAGCAGTGCCTTAGCAGC	GCCAACCTTACTTCAGCAGC	89

miRNAs=MicroRNA

**Supplementary Table-S2:** Oligonucleotide primers for inflammatory and pathogen receptor genes.

mRNA	Accession no.	Forward primer sequence (5′-3′)	Reverse primer sequence (5′-3′)	Products (bp)
*CXCL8*	NM_173925	TCTCTGCAGCTCTGTGTGAAG	TTCCTTGGGGTTTAGGCAGAC	209
*IL1B*	NM_174093	ACAAAAGCTTCAGGCAGGTG	AGCACCAGGGATTTTTGCTC	226
*IL6*	NM_173923	AGCGCATGGTCGACAAAATC	AGCAGTGGTTCTGATCAAGC	179
*TLR4*	AF310952	AATTTGCCAAGGAGCCTCAC	ACAAGTGGCGTTCCTGAAAC	189
*TNF*	NM_173966	AGCACCAAAAGCATGATCCG	TTTGAACCAGAGGGCTGTTG	226
*ACTB*	NM_173979	TGCGGCATTCACGAAACTAC	AGGGCAGTGATCTCTTTCTGC	146

mRNA=Messenger RNA, IL1B=Interleukin 1β, TNF=Tumor necrosis factor, TLR4=Toll-like receptor 4, CXCL8=Chemokine (C-X-C motif) ligand 8, IL6=Interleukin 6, ACTB=Beta-actin

### Real-time polymerase chain reaction (PCR)

Real-time PCR was performed to investigate the effect of quercetin on miRNAs and proinflammatory cytokine gene expression in bovine neutrophils. Analysis of the levels of gene expression was performed in triplicate on an Applied Biosystems 7300 real-time PCR system equipped with SDS software v1.4 (Thermo Fisher Scientific). The real-time PCR was performed using a SensiFAST SYBR^®^ Hi-ROX Kit (Bioline) as per the manufacturer’s instructions. The cycling conditions were set as follows: An initial denaturation of 95°C for 2 min, followed by 40 (mRNAs) or 45 cycles (miRNAs) of denaturation at 95°C for 5 s, and annealing/extension at 60°C for 30 s. Subsequently, specificity was confirmed by dissociation curve analysis (T_m_). Correct product sizes were also determined by 2% agarose gel in 0.5 X tris-acetate-ethylenediaminetetraacetic acid (TAE) buffer at 100 V for 35 min. The gels were stained with ethidium bromide (0.5 µg/mL) and documented using the GelMax Imager (Ultra-Violet Products, Cambridge, UK). Analysis of relative gene expression was calculated from the *C*_T_ of miRNA genes/proinflammatory genes and *MIR16A*/*β-actin* (*ACTB*). The expression levels (fold difference) were reported using the 2^–ΔΔ*C*^_T_ method [23].

### Data analysis

The data were first screened for potential outliers by robust regression and outlier removal method. The normality test was done by the D’Agostino–Pearson omnibus test before performing unpaired two-tailed Student’s *t*-test or Mann–Whitney test. The miRNA expression levels of PBS-treated neutrophils were chosen as the basal level, and the expression levels of quercetin-treated cells were expressed as the relative changing fold compared with the basal level. The statistical analysis was considered significant when p<0.05. The results were reported as mean with standard error (mean±standard error) or median values. The correlation analysis on the differential fold change of genes was performed using psych package in R (programming language, RStudio).

## Results

### Specificity of bovine miRNA and mRNA primers and real-time PCR efficiency

First, we determined the real-time PCR efficiencies with each primer pair of inflammatory miRNAs and cytokine genes by performing standard curve data of log_10_ serially diluted DNA of bovine neutrophils. Coefficient of regression (R^2^) values is outlined in supplementary Table-S3. The R^2^ values for all real-time PCR reactions by regression analysis have values over 0.97 (Supplementary Figures-S1 and S2). The specificity of the primers is presented by Agarose gel electrophoretic images (Supplementary Figures-S3 and S4).

**Supplementary Table-S3:** Standard curve data from real-time PCRs of bovine neutrophils.

Gene	Log dilutions	C_t_	Correlation coefficient (R^2^)	Equation of standard curve
*MIRLET7E*	10^−1^-10^−6^	10-23	0.9996	Y=−2.538*X+25.67
*MIR17*	10^−1^-10^−5^	16-32	0.9929	Y=−4.045*X+37.10
*MIR24-2*	10^−1^-10^−6^	7-23	0.9944	Y=−3.093*X+26.89
*MIR146A*	10^−1^-10^−5^	9-33	0.9972	Y=−5.985*X+39.72
*MIR181C*	10^−1^-10^−6^	8-23	0.9934	Y=−3.013*X+26.65
*MIR16A*	10^−1^-10^−6^	7-24	0.9986	Y=−3.409*X+27.52
*IL1B*	10^−1^-10^−5^	8-20	0.9956	Y=−3.147*X+24.33
*IL6*	10^−1^-10^−5^	10-25	0.9964	Y=−3.759*X+29.47
*CXCL8*	10^−1^-10^−5^	9-21	0.9775	Y=−2.945*X+25.38
*TLR4*	10^−1^-10^−5^	8-24	0.9860	Y=−3.979*X+29.05
*TNF*	10^−1^-10^−5^	10-26	0.9991	Y=−4.100*X+30.73
*ACTB*	10^−1^-10^−5^	11-26	0.9953	Y=−3.611*X+29.76

IL1B=Interleukin 1β, TNF=Tumor necrosis factor, TLR4=Toll-like receptor 4, PCR=Polymerase chain reaction, CXCL8=Chemokine (C-X-C motif) ligand 8, IL6=Interleukin 6, ACTB=Beta-actin

**Supplementary Figure-S1:**
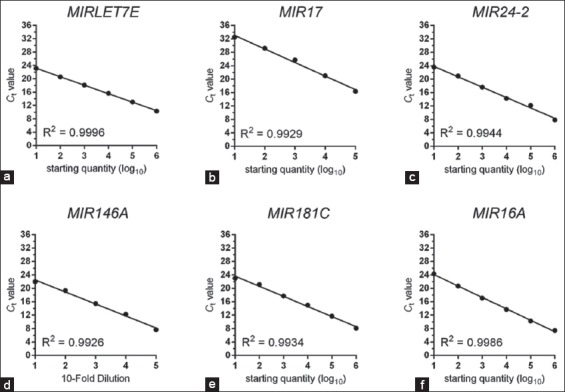
(a-f) Standard curve data of log_10_ serially diluted DNA of selected microRNA genes in bovine neutrophils by real-time polymerase chain reactions (PCRs). Coefficient of regression (R^2^) values are given in each plot. The R^2^ values for all real-time PCR reactions by regression analysis are over 0.99.

**Supplementary Figure-S2:**
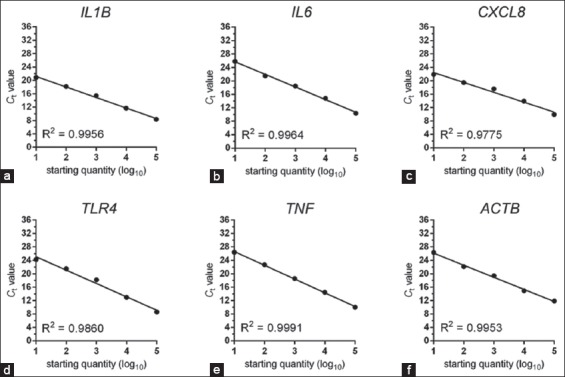
(a-f) Standard curve data of log_10_ serially diluted DNA of selected genes of bovine neutrophils by real-time polymerase chain reactions (PCRs). Coefficient of regression (R^2^) values are given in each plot. The R^2^ values for all real-time PCR reactions by regression analysis are over 0.97.

**Supplementary Figure-S3:**
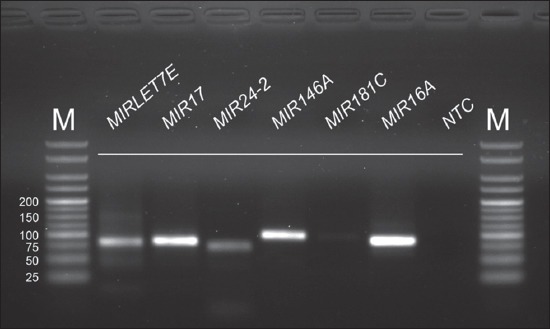
Representative polymerase chain reaction (PCR) products of expressed microRNA genes obtained from bovine neutrophils on agarose gel electrophoresis and EtBr staining after real-time PCRs. All PCR products had the correct size (Supplementary Table-1). *MIR16A* was used as a reference gene. NTC=No template control; M=25 bp DNA marker.

**Supplementary Figure-S4:**
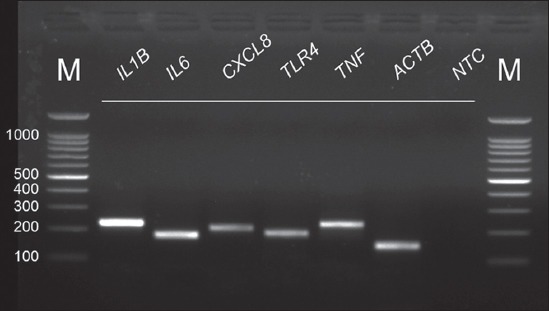
Representative polymerase chain reaction (PCR) products of expressed genes obtained from bovine neutrophils on agarose gel electrophoresis after real-time PCRs. All PCR products had the correct size. *ACTB* was used as a reference gene. NTC=No template control; M=100 bp DNA marker.

### Effects of quercetin on inflammatory miRNA gene expression in bovine neutrophils after LPS stimulation

Previous well-documented data have shown that quercetin significantly inhibits LPS-driven inflammation [16,19-21,24,25]. Here, we demonstrated the effects of quercetin on decreasing inflammatory miRNAs in bovine neutrophils stimulated with LPS. After 60 min incubation, bovine neutrophils were harvested for further examination of inflammatory miRNA gene expression patterns by real-time PCR. As outlined in Table-1, quercetin-treated neutrophils exhibited a remarkable suppression in *MIR24-2*, *MIR146A* and *MIR181C* expression as compared with the untreated cells (Table-1). However, quercetin may be unable to fully attenuate the inflammation mediated by *MIRLET7E* and *MIR17*, as the miRNA expression of these two genes was increased after quercetin treatment.

**Table-1:** Effects of quercetin on the differential expression of inflammatory miRNAs in bovine neutrophils.

miRNAs	PBS	Quercetin	p value
*MIRLET7E*	1.001±0.013 (n=11)	1.171±0.071 (n=11)	0.028
*MIR17*	0.994±0.014 (n=10)	1.185±0.247 (n=10)	0.447
*MIR24-2*	1.000±0.009 (n=12)	0.723±0.118 (n=12)	0.029
*MIR146A*	1.000±0.006 (n=11)	0.784±0.086 (n=11)	0.021
*MIR181C*	1.001±0.004 (n=12)	0.855±0.066 (n=12)	0.037

PBS=Phosphate buffered saline, miRNAs=MicroRNA

### Effects of quercetin on proinflammatory and pathogen receptor gene expression in bovine neutrophils after LPS stimulation

Quercetin clearly possesses an anti-inflammatory property toward proinflammatory cytokine genes in mammalian neutrophils, macrophages, and macrophage cell lines [19,20,24,26,27]. Our initial data indicated that the expression of many proinflammatory cytokine genes and the pathogen receptor *TLR4* in LPS-exposed bovine neutrophils were minimized overall by the action of quercetin. The mRNA expression of *IL1B, IL6, CXCL8, TLR4*, and *TNF* genes noticeably declined in the quercetin group as shown in Table-2. This study provided insightful information on the overall actions of quercetin, i.e., that this substance exerts its anti-inflammatory effects in bovine neutrophil gene expressions.

**Table-2:** Effects of quercetin on the differential expression of proinflammatory mRNAs in bovine neutrophils.

Proinflammatory genes	PBS	Quercetin	p value
*IL1B*	0.999±0.017 (n=11)	0.807±0.114 (n=11)	0.112
*IL6*	0.995±0.015 (n=10)	0.649±0.142 (n=10)	0.026
*CXCL8*	0.977±0.012 (n=10)	0.298±0.063 (n=10)	<0.0001
*TLR4*	0.999±0.010 (n=12)	0.846±0.150 (n=12)	0.317
*TNF*	0.991±0.006 (n=11)	0.618±0.126 (n=11)	0.008

IL1B=Interleukin 1β, TNF=Tumor necrosis factor, TLR4=Toll-like receptor 4, mRNA=Messenger RNA, PBS=Phosphate buffered saline

### Correlation analysis of miRNAs and its cognate mRNA targets in bovine neutrophils

**Figure-1:**
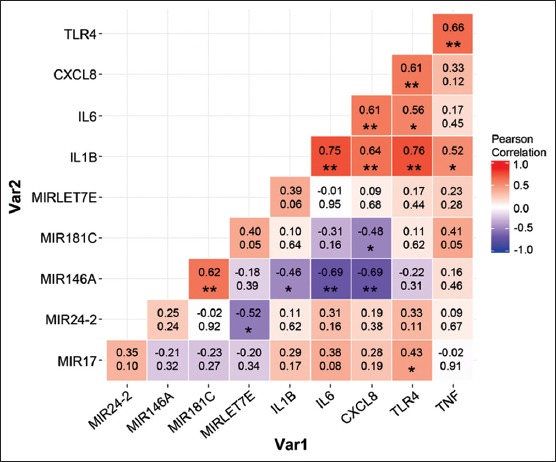
Pearson correlation matrix for comparison of microRNA genes and proinflammatory genes under quercetin supplementation in bovine neutrophils. Numbers in the upper lines on the data matrix are correlation values and numbers in the lower lines are p values (*p<0.05, **p<0.01).

We analyzed the correlations among miRNAs and mRNAs using Pearson correlation to determine whether the effects of quercetin individually contributed to the reduction or elevation of either miRNAs or mRNAs. By coupling the miRNA and its target mRNA, we found that only *MIR146A* and *TLR4* possessed a negative correlation, whereas positive correlations were observed for the rest of the tested genes. We further investigated the relationship among a group of miRNAs and proinflammatory genes by crossing the raw *C*_T_ data in a stepwise manner as shown in data matrices in Figure-1. Interestingly, many proinflammatory genes (*IL1B*, *IL6*, and *CXCL8*) and the pathogen receptor *TLR4* had negative correlations with *MIR146A* and *MIR181C*. Besides *TLR4* and *TNF*, which are postulated to be the targets of *MIR146A* and *MIR181C*, respectively, *IL1B*, *IL6*, and *CXCL8* may also be partially controlled and linked by *MIR146A* and *MIR181C*. As expected, when *MIRLET7E* expression was increased, the expression level of *IL6*, proinflammatory cytokine mRNA, was decreased in parallel. Similar trends were noticeably documented in *MIR17* and *CXCL8* gene expression. On the contrary, *MIR24-2*, *MIR146A*, *MIR181C* and *IL1B*, *TLR4*, *TNF* demonstrated an opposite trend.

## Discussion

The innate immune response to bacterial LPS is multisystem, involved in engaging a pattern recognition receptor, i.e., *TLR4*, and combined responses in both cytokines and chemokines. Our results revealed the expression of cytokine and chemokine genes, as well as a pathogen receptor, in bovine neutrophils after quercetin exposure. As expected, quercetin suppressed the expression of all proinflammatory cytokines and chemokine genes (*IL1B, IL6, CXCL8*, and *TNF*), as well as a selected pathogen receptor (*TLR4*) gene, compared with untreated cells (PBS). These findings are, in part, consistent with results previously reported [16,19,20].

In LPS stimulation and responsiveness in RAW264.7 cells, quercetin may exert its modulatory effects by acting as phosphorylation inhibitors of IκB kinase. The Akt and c-Jun N-terminal kinase are also inhibited by quercetin. Quercetin causes a significant reduction in the phosphorylation and degradation of inhibitor of IκBα and in the nuclear level of nuclear factor-κB, the latter being associated with decreased NF-κB binding activity [28]. It was also shown the anti-inflammatory action of quercetin toward heme oxygenase-1 (HO-1) through NF-κB and STAT1 signaling pathways [29]. Quercetin might alternatively exert its actions by stimulating p38 and then Nrf2, which later activates HO-1 to suppress the formation of reactive oxygen species. Boesch-Saadatmandi *et al*. [24] and Li *et al*. [21] revealed the action of quercetin in suppressing NF-κB, and miR155 whereas stimulating Nrf2 and HO-1, consequently, downregulation of TNF. The solid evidence shows that quercetin manipulated the signaling cascade made by TLR4 stimulation from LPS. The scenarios are the quercetin targets NF-κB before the signaling by TLR4 can be initiated [24]. In addition to targeting this specific transcription factor, quercetin has been reported to target at miR155 for reduced expression of this particular miRNA, subsequently, reduced cognate gene (TNF) of miR155 in the gene expression.

miRNAs are a family of regulatory RNAs that has been reported to be involved in cellular processes in the immune system of cows. A study by Dilda *et al*. challenging bovine monocytes with *E. coli* LPS revealed the upregulation of miR-9, miR-125b, miR-155, miR-146a, and miR-223. These authors claimed that miR-146a is an anti-inflammatory miRNA [22,30]. Our results demonstrated that when isolated bovine neutrophils were supplemented with quercetin and later stimulated by *E. coli* LPS, some miRNA gene expression levels were downregulated, mainly under the control of aforementioned quercetin action, regardless of the inflammatory miRNAs (*MIR24-2*, *MIR181C*) or anti-inflammatory miRNAs (*MIR146A*) [31]. Among many miRNAs, the role of miR-155 is to upregulate proinflammatory cytokines, including TNF-α and IL6, in response to bacterial infection and chronic inflammation [32,33]. According to the study by Dilda *et al.*, the stimulation of bovine monocytes with LPS upregulated both miR-155 and miR-146a [22]. Given the proinflammatory activity of miR-155, upregulation of this miRNA may amplify the proinflammatory loop during the first phase of the innate immune reaction, consistent with the acute inflammatory reaction that follows *E. coli* infection.

The increased expression of *MIRLET7E* and *MIR17* in bovine neutrophils supplemented with quercetin in this study may be because these inflammatory miRNAs are actively involved in LPS-stimulated inflammation at the early phase of infection. Even though the suppression properties of quercetin are actually exerted, higher level of these presenting miRNAs eventually overcame the actions of quercetin. The response of Let-7e and members of let family can modulate the upregulation of *TLR4* expression by functioning as a negative regulator of TLR4 signaling [34-36]. The expression of miRNAs has been linked to ligation of various TLRs and important regulatory roles in innate immune responses [37,38]. Our results showed that the elevation of *MIRLET7E* is conversely related to the decline of *TLR4* expression. Not only quercetin may, in some extent, affect the expression of *TLR4* in an indirect way, but other miRNAs, such as *MIRLET7E* may play some roles in controlling this gene expression.

The function of miR-17, especially miR-17-5p, when in combination with miR-20a and miR-106a is to inhibit monocyte proliferation, differentiation and maturation, as appeared in a review by Lindsay [39]. The transcription of certain miRNAs found in immune cells, such as miR-155 and miR-146a, is upregulated in response to inflammatory stimuli, e.g., TLR ligands or proinflammatory cytokines [33,40]. The exposure to TNF-α and IL1β, or following activation of Toll-like receptor TLR-2, -4, and -5 in macrophages in the alveolar/bronchial epithelium, will induce the expression of miR-146a. The inflammation from a variety of microbial components, such as LPS, revealed that miR-146a is upregulated in *TLR4* stimulation to control NF-κB and other TLR mediators of inflammation [40]. In this regard, miR-146a would be considered as an endotoxin-responsive miRNA. Similarly, the miR-181 family and IL8 are also linked to TLR activation. According to Galicia *et al*. [41] and Zhang *et al.*, [42] the miR-181 family is differentially expressed in the presence of LPS-induced inflammation. In this regard, IL6, IL8, and miR-181 expression were found to have an inversely proportional relationship [41,42]. The overall expression of miRNAs in this study is in accordance with the available information. Pearson linear correlation analysis revealed that a group of proinflammatory cytokine genes, *IL1B*, *IL6*, *CXCL8* and *TNF*, was simultaneously coexpressed in the same trend with the pathogen receptor *TLR4*, as determined by positive correlation. For miRNA gene expression, a positive correlation has been significantly observed between *MIR146A* and *MIR181C*; however, a negative correlation was found between *MIR24-2* and *MIRLET7E*.

## Conclusion

Interaction between cognate mRNAs and miRNAs under quercetin supplementation can be summarized as a positive or negative correlation, as indicated in the data. The expression levels of *MIR146A* were opposed to *IL1B*, *IL6*, and *CXCL8*. The elevated level of *MIR181C* may contribute to the reduction of *CXCL8*. Moreover, the elevation of *MIR17* was found to be positively related to the elevated levels of *TLR4*. This finding may help understand the effects of quercetin either on miRNA or gene expression during inflammation, especially as a potentially applicable indicator in bovine mastitis. However, there are certain limitations in this particular study. To better understand the role of quercetin on miRNA in inflammation, we suggest that miR-155 should be further investigated.

## Authors’ Contributions

PC and SB conceived and designed the study. SS, DS and PC executed the study and involved in data acquisition. SB and PC analyzed and interpreted the data. PC drafted and revised the manuscript. All authors read and approved the final manuscript.
